# Association between maternal obesity and offspring Apgar score or cord pH: a systematic review and meta-analysis

**DOI:** 10.1038/srep18386

**Published:** 2015-12-22

**Authors:** Tingting Zhu, Jun Tang, Fengyan Zhao, Yi Qu, Dezhi Mu

**Affiliations:** 1 Department of Pediatrics, West China Second University Hospital, Sichuan University, Chengdu 610041, China; 2 Key Laboratory of Obstetric & Gynecologic and Pediatric Diseases and Birth Defects of Ministry of Education, Sichuan University, 610041 Chengdu, Sichuan, China; 3 Department of Pediatrics and Neurology, University of California, San Francisco, San Francisco, CA94143, USA

## Abstract

Previous results are inconsistent regarding the association between maternal obesity and Apgar score or cord pH in humans. The aim of this study was to investigate the association between maternal pre-pregnancy and pregnancy body mass index (BMI) and infant Apgar score or cord pH. We conducted a systematic review of studies published in English before 20 August 2015 using PubMed, EMBASE, and Cochrane Library. Eleven cohort studies with a total of 2,586,265 participants finally met our inclusion criteria. Pooled results revealed the following factors associated with Apgar score <7 at 5 minutes: overweight (odds ratio [OR] 1.13; 95% confidence interval [CI], 1.08–1.20), obese (OR 1.40; 95% CI, 1.27–1.54), and very obese (OR 1.71; 95% CI, 1.55–1.89). The pooled analysis also revealed that maternal overweight or obesity increased the risk for Apgar score <7 at 1 minute. There was no association between maternal BMI and neonatal cord pH. Thus, this study suggests that maternal overweight and obesity affect baby’s condition immediately after birth in general. More studies are needed to confirm these results and detect the influence of variables across studies.

The prevalence of female obesity at child-bearing ages has continuously increased in recent years^1^. Maternal baseline body mass index (BMI) values are categorized by standard conventions: underweight (BMI <18.5 kg/m^2^), normal weight (BMI 18.5–24.9 kg/m^2^), overweight (BMI 25–29.9 kg/m^2^), and obese (BMI ≥ 30 kg/m^2^). Maternal obesity is further stratified into three classes: class I (BMI 30–34.9 kg/m^2^), class II (BMI 35–39.9 kg/m^2^), and class III (BMI ≥ 40 kg/m^2^)^2^. Accumulating evidence has suggested that maternal obesity before and during pregnancy can result in pregnancy-induced hypertension, pre-eclampsia, gestational diabetes, and caesarean delivery, as well as foetal and neonatal complications, such as macrosomia, intrauterine growth retardation, foetal death, stillbirth, and infant death^3^,^4^, but the underlying mechanisms have not been well established.

The Apgar score was introduced by Virginia Apgar in 1953 and is evaluated at 1, 5, and 10 minutes after birth. The Apgar score is used as an index to evaluate the neonate’s overall status and response to resuscitation, as well as its prognosis beyond the neonatal period^5^. Furthermore, previous studies have observed that low Apgar scores at birth increased the risk for later motor control and perception difficulties, cognitive developmental delays, learning disabilities, cerebral palsy, autism, attention-deficit/hyperactivity disorder, and epilepsy^6^,^7^.

In addition, cord pH is a more sensitive measure for high risk neonates that may be at risk for poor neurologic outcomes, as most cerebral palsy patients have normal Apgar scores at birth^8^. Cord pH is one assessment of neonatal metabolic status. Cord pH decreases when hydrogen ions from anaerobic metabolism overwhelm the foetus’s buffer capacity, which is an important indicator of birth asphyxia^9^. Birth asphyxia, although the correct definition is imprecise, is an insult to the foetal or newborn due to failure to breath or breathing poorly leading to decrease oxygen perfusion to various organs^10^. It remains a persistent worldwide problem occurring in 20 per 1000 term live births^10^. According to WHO survey, up to 23% of neonatal deaths in low-income countries are due to birth asphyxia^11^. In addition, it is also one of the leading causes of neonatal deaths within 24 hours^12^. Thus, determination of maternal factors that may affect Apgar score and cord pH will improve the understanding of the influence of maternal obesity and provide evidence for predicting serious conditions in order to plan appropriate neonatal care.

To date, many human studies have published inconsistent results on the impact of maternal obesity on Apgar score and cord pH at birth. Thus, we conducted a systematic review and meta-analysis to evaluate their association. We hypothesized that neonates exposed to maternal obesity would have lower Apgar scores and cord pH than would normal controls.

## Results

### Literature search

We identified 205 potential studies: 132 from PubMed, 63 from EMBASE, 4 from the Cochrane Database, and 9 additional references from reviewing references in relevant articles. After careful screening, 11 studies were selected for inclusion in this study^3^,^4^,^13^,^14^,^15^,^16^,^17^,^18^,^19^,^20^,^21^. Supplemental Fig. 1 shows the reasons for exclusion. The extracted data from the 11 included studies are presented in Table 1.

### Characteristics and quality of included studies

The included studies were published between 2008 and 2015. All were cohort studies. The sample sizes varied, from a maximum of 1764403^21^ to a minimum of 1996^4^. Of the included studies, eight selected consecutive singleton births without gestational age restrictions for analysis^3^,^14^,^15^,^16^,^17^,^18^,^19^,^20^, and three were limited to full-term infants^4^,^13^,^21^. Regarding timing of maternal BMI assessment, seven studies assessed BMI at the first antenatal visit^13^,^15^,^17^,^18^,^19^,^20^,^21^, one study at the early second trimester (13–18 weeks)^14^, and three at pre-pregnancy^3^,^4^,^16^. Most studies used normal weight status as the reference, one^14^ used BMI < 25 kg/m^2^, one used non-obese participants^18^, and one used class I obesity^16^.

For data sources of maternal BMI, seven studies used self-reported maternal weight and height^4^,^13^,^15^,^16^,^17^,^18^,^20^,^21^, one was measured by a physician^19^, and three determined BMI from medical records or registry data^3^,^14^,^17^. For Apgar score and cord pH, all studies identified them from registry data or medical records. All studies were controlled statistically for a number of potentially confounding variables. The results of the quality assessment of the included studies are shown in Table 1. All included studies were of high quality (NOS > 5).

### Maternal BMI and Apgar score

**Figure 1:**
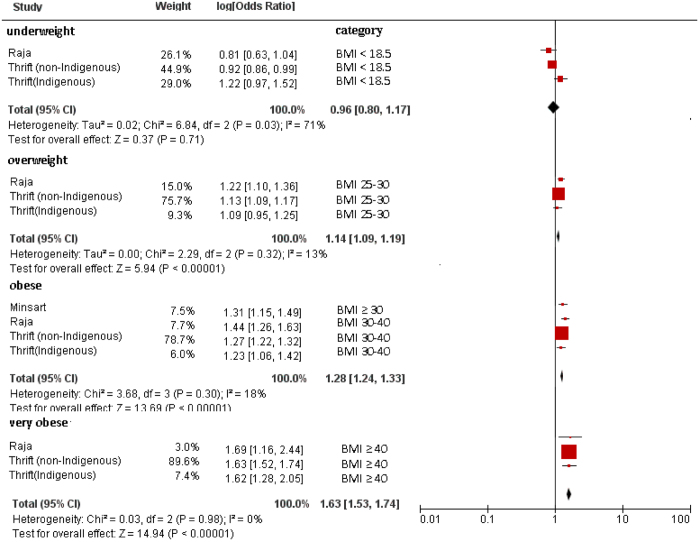
Forest plot of pooled analyses of maternal BMI categories and an Apgar score <7 at 1 minute. Maternal overweight, obesity and very obesity by BMI categories was significantly associated with Apgar score <7. Maternal underweight categories showed nonsignificant trends toward increased an Apgar score <7. Note that obesity group compared with non-obese controls (BMI < 30) in the Minsart *et al.* study. In others studies, ORs are for each category as compared with the “normal weight” category (BMI 18–25).

The original outcomes of the included studies are presented in Table 1. Considering the studies’ heterogeneity, we performed a separate pooled analysis of Apgar scores < 3 at 5 min from those < 7. Compared with infants born to normal-weight mothers, the pooled results of maternal BMI for Apgar scores < 7 at 1 minute were as follows: underweight (odds ratio [OR] = 0.96; 95% confidence interval [CI], 0.80–1.17; *P* = 0.71), overweight (OR = 1.14; 95% CI, 1.09–1.19; *P* < 0.001), obese (OR = 1.28; 95% CI, 1.24–1.33; *P* < 0.001), and very obese (OR = 1.63; 95% CI, 1.53–1.74; *P* < 0.001) (Fig. 1).

**Figure 2:**
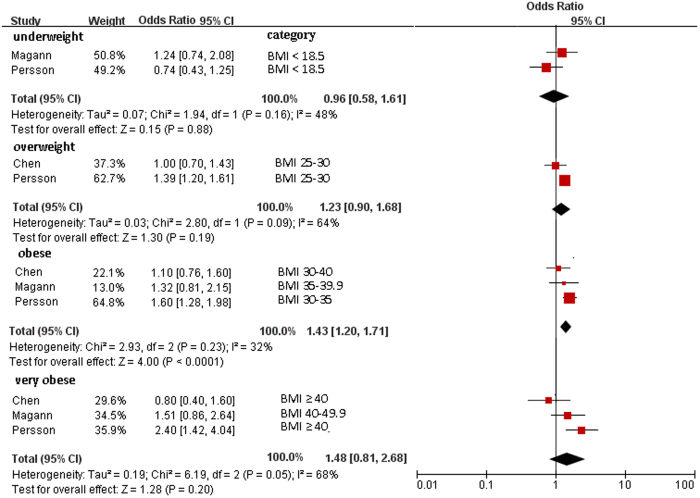
Forest plot of pooled analyses of maternal BMI categories and an Apgar score <3 at 5 minute. Maternal obesity by BMI categories was significantly associated with Apgar score <3. Maternal underweight, overweight, and very obesity categories showed nonsignificant trends toward increased an Apgar score <3. Note that ORs are for each category as compared with different reference category (Chen *et al.*, BMI < 25; Magann *et al.*, BMI 18–25; Persson *et al.*, BMI 18.5–34.9).

Figure 2 shows the association between maternal BMI and Apgar score < 3 at 5 minutes: underweight (OR = 0.96; 95% CI, 0.58–1.61; *P* = 0.88), overweight (OR = 1.23; 95% CI, 0.90–1.68; *P* = 0.19), obese (OR = 1.43; 95% CI, 1.20–1.71; *P* < 0.001), and very obese (OR = 1.48; 95% CI, 0.81–2.68; *P* = 0.20).

**Figure 3:**
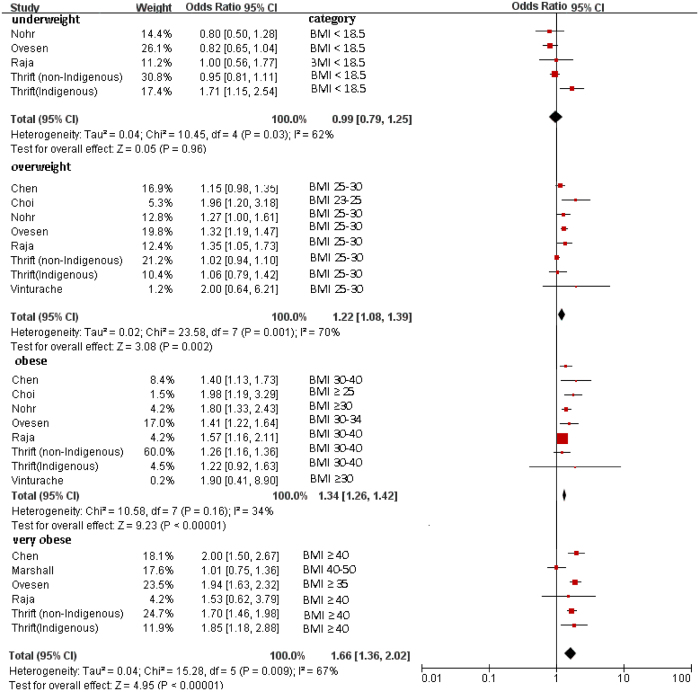
Forest plot of pooled analyses of maternal BMI categories and an Apgar score <7 at 5 minute. Maternal overweight, obesity and very obesity by BMI categories was significantly associated with Apgar score <7. Maternal underweight categories showed nonsignificant trends toward increased an Apgar score <7. Note that reference category was BMI < 25 in Chen *et al.* study. In other studies, ORs are for each category as compared with the “normal weight” category (BMI 18–25).

Maternal BMI showed significant trends for Apgar scores <7 at 5 minutes: underweight (OR = 0.99; 95% CI, 0.79–1.25; *P* = 0.96), overweight (OR = 1.22; 95% CI, 1.08–1.39; *P* = 0.002), obese (OR = 1.34; 95% CI, 1.26–1.42; *P* < 0.001), and very obese (OR = 1.66; 95% CI, 1.36–2.02; *P* < 0.001) (Fig. 3).

We identified one study^21^ that reported a significant association between Apgar score <4 at 10 minutes and maternal overweight or obesity (BMI 25–29.9: 1.32 (1.10–1.58); BMI 30–34.9: 1.57 (1.20–2.07); BMI 35–39.9: 1.80 (1.15–2.82); and BMI 40: 3.41 (1.91–6.09)).

### Maternal BMI and cord pH

**Figure 4:**
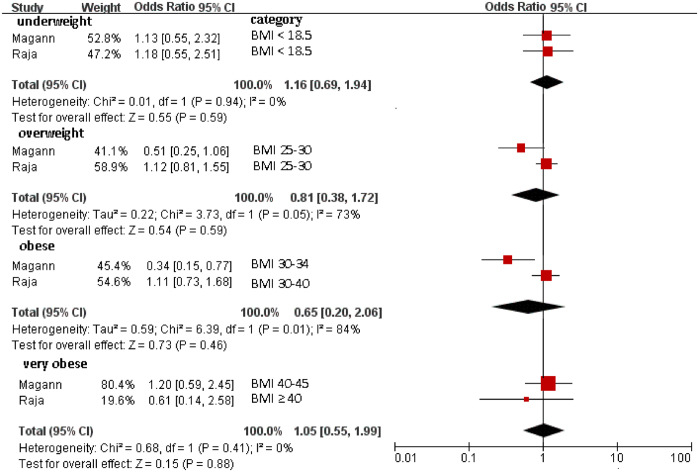
Forest plot of pooled analyses of maternal BMI categories and cord pH < 7.1. Maternal BMI in different categories showed nonsignificant associations with cord pH < 7.1. Note that ORs are for each category as compared with the “normal weight” category (BMI 18–25).

Two studies reported ORs by BMI categories for cord pH < 7.1 18,20. The pooled analysis showed no significant association with maternal BMI: underweight (OR = 1.16; 95% CI, 0.69–1.94; *P* = 0.59), overweight (OR = 0.81; 95% CI, 0.38–1.72; *P* = 0.59), obese (OR = 0.65; 95% CI, 0.20–2.06; *P* = 0.46), and very obese (OR = 1.05; 95% CI, 0.55–1.99; *P* = 0.88) (Fig. 4).

### Publication bias

A funnel plot was used for visual assessment of publication bias only when at least 10 studies were included in the meta-analysis. Thus, an adjusted Begger’s test was implemented to evaluate asymmetry and publication bias. The pooled results showed no evidence of publication bias (supplemental Table 6).

### Subgroup analysis and sensitivity analysis

We carried out a subgroup analysis for pooled results if at least two studies existed for each group. For Apgar score < 7 at 5 minutes, a higher OR was observed in studies including only full-term infants (1.80, 95% CI, 1.33–2.43; *P* < 0.001) than in other studies (1.32, 95% CI, 1.24–1.40; *P* < 0.001) for analyses of the association between Apgar score and obese mothers. For timing of measurement of maternal BMI, there was a higher OR in studies with pre-pregnancy BMI assessment (1.97, 95% CI 1.22–3.20; *P* = 0.006) than in others (1.33, 95% CI 1.25–1.41; *P* < 0.001) for the association with obese mothers. These results are similar to those for the association with overweight mothers (for more details, see the supplemental Table 5).

Considering the small number of studies in our pooled analysis, we performed a sensitivity analysis for Apgar score <7 at 5 minutes by omitting one study at a time. Every BMI category maintained similar results during the sensitivity analysis. For underweight, significant heterogeneity disappeared (*P* = 0.7; I^2^ = 0%) when excluding the outcomes related to indigenous pregnancy from the Thrift *et al.* study^20^. For overweight, heterogeneity decreased to 68% (*P* = 0.004), 58% (*P* = 0.03), and 18% (*P* = 0.29), when the Choi *et al.*^3^, Ovesen *et al.*^15^, and non-indigenous Thrift *et al.*^20^ studies were excluded, respectively. For obesity, heterogeneity decreased to 27% (*P* = 0.22) and 9% (*P* = 0.36) when the Choi *et al.*^3^ and Nohr *et al.*^13^ studies were excluded, respectively. In addition, no evidence of heterogeneity was observed among the remaining studies when the outcomes related to non-indigenous pregnancy in Thrift *et al.* study^20^ were excluded (*P* = 0.16, I^2^ = 0%). For the very obese category, heterogeneity decreased (*P* = 0.77, I^2^ = 0%) only when the Marshall *et al.*^16^ study was excluded.

## Discussion

The results of this systematic review and meta-analysis of 11 cohort studies with a total of 2,586,265 participants showed that infants whose mothers had a BMI ≥ 25 kg/m^2^ during pregnancy had an increased risk of low Apgar scores at 1 and 5 minutes. Maternal underweight (defined by BMI) was not associated with low Apgar scores. Maternal BMI was not associated with cord pH. However, these pooled results may have been underpowered because of the small number of studies included. The subgroup analysis and sensitivity analysis showed the stability of pooled results.

The Apgar score at 5 minutes was shown to be more predictive of neonatal survival than that at 1 minute^22^. Low Apgar score at 5 minutes was associated with an increased risk of neonatal and infant death, with a higher magnitude for very low Apgar (0–3) compared with intermediate scores (4–6). Furthermore, the strength of these association was strongest for full-term infants^23^. Previous studies have observed that maternal BMI was related to risks of infant mortality primarily in full-term births^24^. Our study suggested that maternal obesity was associated with low Apgar scores at 1 and 5 minutes. In a subgroup analysis, the risk of maternal obesity and overweight was higher for Apgar score <7 at 5 minutes in full-term infants and time of measurement of maternal BMI at pre-pregnancy. However, these two groups contained overlapping studies, so it cannot be concluded that a high BMI assessed pre-pregnancy is more harmful than that assessed during early pregnancy. Also, weight gain during pregnancy was associated with neonatal adverse events^25^,^26^. Of the included studies, more than half of studies assessed maternal BMI at the first antenatal visit without exact time. Thus some women may receive their first check-up in the middle or late stage of pregnancy, and gain certain weights by her pregnancy. Future studies are warranted to detect the influence of these variables.

Cord pH is considered a crucial outcome measure for monitoring foetal conditions. Malin *et al.* concluded that low arterial pH was strongly associated with long-term adverse outcomes in a systematic review of outcomes for 481,753 infants^27^. However, the clinically meaningful pH level is unknown. Yeh *et al.* suggested that the threshold pH for adverse neurological outcomes is 7.10 and the ‘ideal’ cord pH is 7.26–7.30. Above 7.00, however, neonatal acidemia is weakly associated with adverse outcomes^28^. Our results did not show any association between umbilical cord pH < 7.1 and maternal BMI. However, measurement of umbilical cord pH is not part of routine care in some obstetrics facilities, so we only had two applicable studies. Heterogeneity existed among these studies. Further, the lack of other cut-off criteria besides pH < 7.1 for cord pH might explain the absence of an association. Thus, more clinical studies are required to assess the relationship between arterial umbilical cord pH and maternal BMI.

Maternal obesity might affect the neonatal condition immediately after birth through multiple pathways. Many studies have demonstrated that obesity in pregnancy is associated with a wide spectrum of maternal complications, including postpartum haemorrhage, higher risks of maternal hypertension, and gestational diabetes. Obesity in pregnancy has also been shown to be associated with foetal macrosomia, post-term pregnancy, increased caesarean section rates, and need for labour induction^25^,^26^,^29^.

Maternal BMI in early pregnancy is strongly associated with fat mass, which includes visceral fat mass. The placenta is prone to obesity-associated lipid accretion^30^,^31^. Previous studies have observed that maternal obesity is associated with elevated total cholesterol, low-density lipoprotein cholesterol, very low-density lipoprotein cholesterol, triglycerides, and lower high-density lipoprotein cholesterol^32^. Placentas from obese women show 50% more lipids than do placentas from lean women^33^. Alternatively, lipotoxicity may influence the pathogenesis of the placenta through inflammation and oxidative stress. Compared with full-term control placentas, placentas of obese women showed disorders of redox balance, as indicated by increased lipid peroxidation (malondialdehyde measurement) and activity of antioxidant enzymes, such as the superoxide dismutases, catalase, and glutathione peroxidase^34^. Finally, increasing lipotoxicity, inflammation, and oxidative stress in the placenta could disrupt placental morphology, cell proliferation, and angiogenesis^32^. Placental dysfunction could impair fetal health condition in the uterus.

Substantial heterogeneity was observed in the pooled analysis, but this was not surprising because of the limited number of included studies and differences in various aspects across studies. The sensitivity analysis suggested that the heterogeneity might come from specific studies. In the Choi *et al.* study^3^, low Apgar scores were defined as scores less than 7 at 1 or 5 minutes, which may lead to overestimated results. In fact, the OR reported by that study was evidently higher than that in the other studies. Similarly, Nohr *et al.*^13^ included Apgar scores <8, which might account for their results. Marshall *et al.*^16^ reported ORs for very obese which was compared to mothers BMI ranged from 30 to 39.9. In addition to differences in the features of the study populations, Thrift *et al.*^20^ conducted a study with indigenous or non-indigenous pregnancies, which contributed to the high heterogeneity. The ethnicity basis for association between overweight or obesity and adverse neonatal outcome remain unclearly.

One of major strengths of our study is that all included original studies used a cohort design, eliminating the possibility of reverse causation. Moreover, in the sensitivity analysis, the combined results of the associations between maternal overweight and obesity with the risk of low Apgar scores persisted and remained statistically significant. In addition, with the large sample size, we had enhanced statistical power to provide more precise and reliable risk estimates. The major limitation of our meta-analysis was that currently available published studies in this area are not sufficient. Additionally, we included only articles published in English. For studies on maternal BMI, cord pH, and Apgar scores at 1 minute, only 2 to 3 studies were eligible for analysis under each category.

In conclusion, our pooled analyses provide evidence that maternal overweight and obesity are significantly associated with low Apgar scores. There was no association with low cord pH. However, the associations could not be definitively concluded to indicate risk factors. More studies are needed to focused on this topic.

## Methods

### Retrieval of studies

We searched PubMed, EMBASE, and Cochrane Library in order to located related studies. The literature search was completed before August 2015. The search was performed by combining Medical Subject Heading (MeSH) terms combined with free-text words for birth asphyxia, such as asphyxia neonatorum and Apgar score, or keywords, such as birth asphyxia and cord pH, and using “OR” for connecting relevant text within the concept. To acquire studies related to maternal weight status we combined these terms using “AND” with a combination of key word, such as maternal obesity, maternal body mass index, and gestational weight. We restricted the search to human studies published in English. Titles and abstracts of the retrieved studies were scanned to exclude studies that were clearly irrelevant. Then, two authors independently read the full text of remaining studies to determine their eligibility according to our inclusion criteria. Disagreements were resolved by a third author, who independently examined the studies, and then consensus was reached. The reference lists of the included studies and relevant reviews were manually searched for further additional articles.

### Study selection

Inclusion criteria for our study were as follows: (1) those that investigated maternal BMI during pregnancy and risk of low Apgar score and cord pH; (2) case-control or cohort studies; (3) those that described the assessment of exposure and outcome; and (4) those that provided adjusted relative risk (RR) estimates, such as risk ratios, incidence rate ratios, hazard ratios, or ORs and 95% CIs for different categories of BMI.

Exclusion criteria for the study were as follows: (1) case series; (2) studies with overlapping data; (3) those that provided crude ORs or original data; (4) those in which Apgar scores and cord pH were expressed as means and standard deviations.

### Data extraction

Two investigators independently extracted data from the studies, including first author, publication date, country, study design, sample size, assessment method for maternal BMI and birth asphyxia parameters, primary outcome, and adjusted confounders. We included the single study with the largest sample size if participants overlapped between studies. When data extraction was completed, we compared the results of two authors, and any disagreement was independently reviewed by a third author until consensus was reached.

### Quality evaluation

All included studies were examined for their methodological quality by two authors independently using the Newcastle-Ottawa scale (NOS). The NOS is recommended for both cohort studies and case-control studies, where scores vary between 0 and 9^35^. This scale consists of a eight-term process for assessing the selection of the study population, comparability, and the evaluation of exposure and outcome. Studies with scores of at least 5 were deemed to be high-quality studies. Any disagreement was resolved in the manner previously described.

### Statistical analysis

Included studies used ORs to assess the association between maternal BMI and risk of low Apgar score and cord pH. We pooled the ORs across studies using the Mantel–Haenszel formula (fixed-effect model) or the DerSimonian–Laird formula (random-effect model). A fixed-effect model was adopted when heterogeneity existed; otherwise, a random-effect model was used. The I^2^ and Q statistics were used to detect statistical heterogeneity between studies. The Q statistic was considered significant if *P* < 0.1, and I^2^ > 50% indicated high heterogeneity. A forest plot was used to show the ORs and 95% CIs for each study, as well as the pooled ORs and 95% CIs. We conducted subgroup analyses in studies where participants were restricted based on gestational age (full-term infant or not) and time of measurement of maternal BMI (pre-pregnancy or during pregnancy). We performed the sensitivity analysis by removing one study at a time. Publication bias was visually assessed with funnel plots and the Begg-adjusted rank correlation test, where a value of *P* < 0.05 was considered statistically significant. All analyses wwereas performed with Review Manager software (RevMan, version 5.3) or Stata software (version 12).

## Additional Information

**How to cite this article**: Zhu, T. *et al.* Association between maternal obesity and offspring Apgar score or cord pH: a systematic review and meta-analysis. *Sci. Rep.* **5**, 18386; doi: 10.1038/srep18386 (2015).

## Supplementary Material

Supplementary Information

## Figures and Tables

**Table 1:** Characteristics of Included Studies.

Author, year	country	Study design	size	GA	Ascertainment ofExposure; Outcome	Primary outcome	Comments	Risk ofbias;quality
Nohr 2008^13^	Danish	Retrospective cohort	58126	term	Self-report; registry data	Apgar score (<8) at 5 min BMI < 18.5, OR = 0.8 (CI, 0.5–1.3) BMI 25–30, OR = 1.3 (CI,1.0–1.6) BMI ≥ 30, OR = 1.8 (CI, 1.3–2.4)	GWG, maternal age, parity, height, smoking, alcohol consumption, social status, exercise, GA, and BW	Different methods of exposure, only preterm neonates; NOS:6
CHEN 2010^14^	United States	retrospective cohort	58089	all	Registry data	Apgar score (4–6) at 5min BMI 25–30, OR = 1.2 (CI, 0.99–1.4) BMI 30–40, OR = 1.4 (CI, 1.1–1.7) BMI ≥ 40, OR = 2.0 (CI, 1.5–2.7) Apgar score (0–3) at 5min BMI 25–30, OR = 1.0 (CI, 0.7–1.4) BMI 30–40, OR = 1.1 (CI, 0.8–1.6) BMI ≥ 40, OR = 0.8 (CI, 0.4–1.6)	Maternal age, maternal education, smoking, preexisting diabetes mellitus, chronic hypertension, maternal fever at delivery, parity, previous preterm or small–for gestational age newborn, birth year	Different methods of exposure; NOS:8
Choi 2011^3^	Korean	retrospective cohort	2454	all	Medical record	Apgar score (<7) at 1 min or 5 min BMI < 18.5, OR = 1.39 (CI, 0.91–2.12) BMI 23–25, OR = 1.96 (CI, 1.20–3.18 BMI ≥ 25, OR = 1.98 (CI, 1.19–3.29)	Maternal age, parity, numbers of fetuses, GA, and medical history	Different methods of exposure; NOS:8
Ovesen 2011^15^	Danish	retrospective cohort	369347	all	Self–report; registry data	Apgar score < 7 at 5 min BMI < 18.5, OR = 0.8 (CI,0.65–1.04) BMI25–29, OR = 1.32 (CI,1.19–1.47) BMI 30–34,OR = 1.41 (CI,1.22–1.64) BMI ≥ 35, OR = 1.94 (CI,1.63–2.32)	Maternal age, parity, smoking during pregnancy, GA, BW, gestational diabetes mellitus, sex, and birth year	Different methods of exposure; NOS:7
Marshall 2012^16^	United States	retrospective cohort	64272	all	Self–report; medical record	Apgar score < 7 at 5 min BMI 40–49.9, OR = 1.0 (CI, 0.8–1.4) BMI ≥ 50, OR = 1.9 (CI, 1.1–3.2)	smoking, medicaid, age (18–34 years), education, prenatal care, married, nulliparous, repeat cesarean delivery, scheduled primary cesarean delivery, and race	Different methods of exposure; NOS:7
Raja 2012^17^	United Kingdom	retrospective cohort	27668	all	Medical record	Apgar score < 8 at 1 min BMI < 18.5, OR = 0.81 (CI,0.63–1.04) BMI 25–30, OR = 1.22 (CI,1.10–1.36) BMI 30–40, OR = 1.44 (CI,1.26–1.63) BMI ≥ 40, OR = 1.69 (CI,1.16–2.44) Apgar score < 8 at 5 min BMI < 18.5, OR = 1.00 (CI,0.56–1.77) BMI 25–30, OR = 1.35 (CI,1.06–1.73) BMI 30–40, OR = 1.57 (CI,1.16–2.11) BMI ≥ 40, OR = 1.53 (CI,0.62–3.79) Cord pH < 7.10 BMI < 18.5, OR = 1.18 (CI, 0.55–2.51) BMI 25–30, OR = 1.12 (CI, 0.81––1.55) BMI 30–40, OR = 1.11 (CI, 0.73–1.68) BMI ≥ 40, OR = 0.61 (CI, 0.14–2.58)	maternal age, ethnicity, parity and smoking	Different methods of exposure; NOS:8
Minsart 2013^18^	Belgian	Retrospective cohort	38675	all	Registry data or self–report; registry data	Apgar score < 7 at 1 min BMI ≥ 30, OR = 1.31 (CI, 1.15–1.49)	maternal age, parity, GWG, height, multiple birth, hypertension, diabetes, macrosomia, gestational age, maternal origin, education, employment, cohabiting status	Different methods of exposure; NOS:7
MAGANN 2013^19^	United States	Prospective cohort	4490	all	Measured; medical data	Apgar score (0–4) at 5 min BMI < 18.5, OR = 1.24 (CI, 0.75–2.08) BMI 35–39.9, OR = 1.32 (CI, 0.81–2.15) BMI 40–44.9, OR = 1.51 (CI, 0.86–2.64) BMI ≥ 45, OR = 0.97 (CI, 0.51–1.85) Cord pH < 7.1 BMI < 18.5, OR = 1.13 (CI, 0.56–2.37) BMI 25–29.9, OR = 0.51 (CI, 0.30–1.07) BMI 30–34.9, OR = 0.34 (CI, 0.15–0.77) BMI 35–39.9, OR = 0.55 (CI, 0.25–1.21) BMI 40–44.9, OR = 1.20 (CI, 0.59–2.45) BMI ≥ 45, OR = 1.40 (CI, 0.74–2.77)	maternal age, ethnicity, nullparity, pre-existing hypertension, pre-existing diabetes, induction of labour, caesarean delivery, gestational age, post-term delivery, caesarean delivery, meconium, shoulder dystocia	Different methods of exposure; NOS:8
Thrift 2014^20^	Australia	Retrospective cohort	37752	all	Self-report; registry data	Apgar score < 7 at 1 min Indigenous BMI < 18.5, OR = 1.22 (CI, 0.97–1.52) BMI 25–30, OR = 1.09 (CI, 0.95–1.25) BMI 30–39.9, OR = 1.23 (CI, 1.06–1.42) BMI ≥ 40, OR = 1.62 (CI, 1.28–2.05) Non–indigenous BMI < 18.5, OR = 0.92 (CI, 0.86–0.99) BMI 25–30, OR = 1.13 (CI, 1.09–1.17) BMI 30–39.9, OR = 1.27 (CI, 1.22–1.32) BMI ≥ 40, OR = 1.63 (CI, 1.52–1.74) Apgar score < 7 at 5 min BMI < 18.5, OR = 1.71 (CI, 1.15–2.54) BMI 25–30, OR = 1.06 (CI, 0.80–1.42) BMI 30–39.9, OR = 1.22 (CI, 0.92–1.63) BMI ≥ 40, OR = 1.85 (CI, 1.18–2.88) Non-indigenous BMI < 18.5, OR = 0.95 (CI, 0.81–1.11) BMI 25–30, OR = 1.02 (CI, 0.94–1.10) BMI 30–39.9, OR = 1.26 (CI, 1.16–1.36) BMI ≥ 40, OR = 1.70 (CI, 1.46–1.98)	maternal age, nulliparity Accessibility/Remoteness Index of Australia category and smoking status.	Different methods of exposure; NOS:7
Persson 2014^21^	Sweden	Prospective cohort	1764403	term	Self-report; registry data	Apgar score (0–3) at 5 min BMI < 18.5, OR = 0.74 (CI, 0.43–1.25) BMI 25–30, OR = 1.39 (CI, 1.20–1.61) BMI 30–35, OR = 1.60 (CI, 1.28–1.98) BMI 35–40, OR = 1.61 (CI, 1.11–2.34) BMI ≥ 40, OR = 2.40 (CI, 1.42–4.04) Apgar score (0–3) at 10 min BMI < 18.5, OR = 0.91 (CI, 0.52–1.59) BMI 25–30, OR = 1.28 (CI, 1.07–1.54) BMI 30–35, OR = 1.42 (CI, 1.06–1.89) BMI 35–40, OR = 1.68 (CI, 1.04–2.72) BMI ≥ 40, OR = 3.30 (CI, 1.80–6.03)	maternal country of birth, smoking in early pregnancy, education, parity, height, maternal age, infant year of birth, and mode of delivery	Different methods of exposure, only preterm neonates; NOS:6
Vinturache 2015^4^	Canada	prospective cohort	1996	term	Self-report; medical records	Apgar score < 7 at 5 min BMI 25–30, OR = 2.0 (CI, 0.6–6.2) BMI ≥ 30, OR = 1.9 (CI, 0.4–8.9)	pregnancy complications, type of labour onset, mode of delivery, and meconium in the amniotic fluid	Different methods of exposure, only preterm neonates; NOS:6

GWG, gestational weight gain; GA, gestational age; BW, birth weight; BMI, body mass index; OR, odds ratios; NOS, score of Newcastle-Ottawa scale; min, minute.

## References

[b1] Vahratian A. Prevalence of overweight and obesity among women of childbearing age: results from the 2002 National Survey of Family Growth. J. Maternal Child Health. 13, 268–273 (2009). 10.1007/s10995-008-0340-6 PMC2635913 18415671

[b2] World Health Organization (WHO). Obesity and overweight: WHO. Fact sheet no. 311 (2013). Available at: www.who.int/mediacentre/ factsheets/fs311/en/ (Accessed April 17, 2014).

[b3] Choi S. K., Park I. Y. & Shin J. C. The effects of pre-pregnancy body mass index and gestational weight gain on perinatal outcomes in Korean women: a retrospective cohort study. Reprod Biol Endocrinol. 9, 6 (2011). 21241516 10.1186/1477-7827-9-6 PMC3033321

[b4] Vinturache A. E., McDonald S., Slater D. & Tough S. Perinatal outcomes of maternal overweight and obesity in term infants: a population-based cohort study in Canada. Sci Rep. 5, 9334 (2015). 25791339 10.1038/srep09334 PMC4366803

[b5] Sekhavat L. & Fallah R. Could maternal pre-pregnancy body mass index affect Apgar score? Arch Gynecol Obstet. 287, 15–18 (2013). 22878907 10.1007/s00404-012-2503-3

[b6] Stuart A., Otterblad O. P. & Källen K. Apgar scores at 5 minutes after birth in relation to school performance at 16 years of age. Obstet Gynecol. 118, 201–208 (2011). 21734618 10.1097/AOG.0b013e31822200eb

[b7] Krebs L., Langhoff-Roos J. & Thorngren-Jerneck K. Long-term outcome in term breech infants with low Apgar score—a population-based follow-up. Eur J Obstet Gynecol Reprod Biol. 100, 5–8 (2001). 11728648 10.1016/s0301-2115(01)00456-0

[b8] Vukojevic M., Soldo I. & Granic D. Risk factors associated with cerebral palsy in newborns. Coll Antropol. 33, 199–201 (2009). 20120414

[b9] Tuuli M. G., Stout M. J., Shanks A., Odibo A. O., Macones G. A. & Cahill A. G. Umbilical Cord Arterial Lactate Compared With pH for Predicting Neonatal Morbidity at Term. Obstet Gynecol. 124, 756–761 (2014). 25198278 10.1097/AOG.0000000000000466 PMC4379505

[b10] Aslam H. M. *et al.* Risk factors of birth asphyxia. Ital J Pediatr. 40, 94 (2014). 25526846 10.1186/s13052-014-0094-2 PMC4300075

[b11] Yeh P., Emary K. & Impey L. The relationship between umbilical cord arterial pH and serious adverse neonatal outcome: analysis of 51,519 consecutive validated samples. BJOG. 119, 824–831 (2012). 22571747 10.1111/j.1471-0528.2012.03335.x

[b12] Bryce J., Boschi-Pinto C., Shibuya K., Black R. E. & WHO. Child Health Epidemiology Reference Group. WHO estimates of the causes of death in children. Lancet. 365, 1147–1152 (2005). 15794969 10.1016/S0140-6736(05)71877-8

[b13] Nohr E. A. *et al.* Combined associations of prepregnancy body mass index and gestational weight gain with the outcome of pregnancy. Am J Clin Nutr. 87, 1750–1759 (2008). 18541565 10.1093/ajcn/87.6.1750

[b14] Chen M. *et al.* Maternal obesity and neonatal Apgar scores. J Matern Fetal Neonatal Med. 23, 89–95 (2010). 19670044 10.3109/14767050903168440

[b15] Ovesen P., Rasmussen S. & Kesmodel U. Effect of prepregnancy maternal overweight and obesity on pregnancy outcome. Obstet Gynecol. 118, 305–12 (2011). 21775846 10.1097/AOG.0b013e3182245d49

[b16] Marshall N. E. *et al.* Maternal superobesity and perinatal outcomes. Am J Obstet Gynecol. 206, 417.e 1–6 (2012). 22542116 10.1016/j.ajog.2012.02.037 PMC3516385

[b17] Raja U. A., Mcaree T., Bassett P. & Sharma S. The implications of a raised maternal BMI: a DGH experience. J Obstet Gynaecol. 32, 247–251 (2012). 22369397 10.3109/01443615.2011.645920

[b18] Minsart A. F., Buekens P., De Spiegelaere M. & Englert Y. Neonatal outcomes in obese mothers: a population-based analysis. BMC Pregnancy Childbirth. 13, 36 (2013) 23398843 10.1186/1471-2393-13-36 PMC3575268

[b19] Magann E. F. *et al.* The effects of an increasing gradient of maternal obesity on pregnancy outcomes. Aust N Z J Obstet Gynaecol. 53, 250–257 (2013). 23432797 10.1111/ajo.12047

[b20] Thrift A. P. & Callaway L. K. The effect of obesity on pregnancy outcome among Australian Indigenous and non-Indigenous women. Med J Aust. 201, 592–595 (2014). 25390266 10.5694/mja13.11170

[b21] Persson M., Johansson S., Villamor E. & Cnattingius S. Maternal overweight and obesity and risks of severe birth-asphyxia-related complications in term infants: a population-based cohort study in Sweden. PLoS Med. 11, e1001648 (2014). 24845218 10.1371/journal.pmed.1001648 PMC4028185

[b22] Sykes G. S. *et al.* Do Apgar score Indicate asphyxia? Lancet. 319, 494–496 (1982). 6121150 10.1016/s0140-6736(82)91462-3

[b23] Iliodromiti S. *et al.* Apgar score and the risk of cause-specific infant mortality: a population-based cohort study. Lancet. 384, 1749–1755 (2014). 25236409 10.1016/S0140-6736(14)61135-1

[b24] Johansson S. *et al.* Maternal overweight and obesity in early pregnancy and risk of infant mortality: a population based cohort study in Sweden. BMJ. 349, g6572 (2014). 25467170 10.1136/bmj.g6572 PMC4252825

[b25] Mochhoury L., Razine R., Kasouati J., Kabir I M. & Barkat A. Body Mass Index, Gestational Weight Gain, and Obstetric Complications in Moroccan Population. J Pregnancy. 2013, 379461 (2013). 23936654 10.1155/2013/379461 PMC3723322

[b26] Chen Z., Du J., Shao L., Zheng L., Wu M., Ai M. & Zhang Y. Prepregnancy body mass index, gestational weight gain, and pregnancy outcomes in China. J Pregnancy. 109, 41–44 (2010). 10.1016/j.ijgo.2009.10.015 20018282

[b27] Malin G. L., Morris R. K. & Khan K. S. Strength of association between umbilical cord pH and perinatal and long term outcomes: systematic review and meta-analysis. BMJ. 340, c1471 (2010). 20466789 10.1136/bmj.c1471 PMC2869402

[b28] Yeh P., Emary K. & Impey L. The relationship between umbilical cord arterial pH and serious adverse neonatal outcome: analysis of 51,519 consecutive validated samples. BJOG. 119, 824–831 (2012). 22571747 10.1111/j.1471-0528.2012.03335.x

[b29] Wahabi H. A., Fayed A. A., Alzeidan R. A. & Mandil A. A. The independent effects of maternal obesity and gestational diabetes on the pregnancy outcomes. BMC Endocr Disord. 13, 47 (2014). 24923207 10.1186/1472-6823-14-47 PMC4065087

[b30] Sewell M. F., Huston-Presley L., Super D. M. & Catalano P. Increased neonatal fat mass, not lean body mass, is associated with maternal obesity. Am J Obstet Gynecol. 195, 1100–1103 (2006). 16875645 10.1016/j.ajog.2006.06.014

[b31] Soltani H. & Fraser R. B. A longitudinal study of maternal anthropometric changes in normal weight, overweight and obese women during pregnancy and postpartum. Br J Nutr. 84, 95–101 (2000). 10961165 10.1017/s0007114500001276

[b32] Scifres C. M., Catov J. M. & Simhan H. N. The impact of maternal obesity and gestational weight gain on early and mid-pregnancy lipid profiles. Obesity (Silver Spring). 22, 932–938 (2014). 23853155 10.1002/oby.20576 PMC4362720

[b33] Saben J. *et al.* Maternal obesity is associated with a lipotoxic placental environment. Placenta. 35, 171–177 (2014). 24484739 10.1016/j.placenta.2014.01.003 PMC3978121

[b34] Malti N. *et al.* Oxidative stress and maternal obesity: feto-placental unit interaction. Placenta. 35, 411–416 (2014). 24698544 10.1016/j.placenta.2014.03.010

[b35] Wells G. *et al.* The Newcastle-Ottawa Scale (NOS) for assessing the quality of nonrandomised studies in meta-analyses. (2011) Available at:http://www.ohri.ca/programs/clinical_epidemiology/oxford.asp (Date of access: 4th July 2014).2014>

