# Supplementary material for: Association between maternal obesity and offspring Apgar score or cord pH: a systematic review and meta-analysis
Source: Sci Rep. 2015 Dec 22;5:18386. doi: 10.1038/srep18386 (PMC4686911; doi:10.1038/srep18386)
Supplement: Supplementary Information [file srep18386-s1.doc]

**Supplementary Data**

Association between maternal obesity and offspring Apgar score or cord pH: a systematic review and meta-analysis. Tingting Zhu, Jun Tang, Fengyan Zhao, Yi Qu, Dezhi Mu.

| sTable 1. Search Strategy in PubMed | 2 |
| --- | --- |
| sTable 2. Search Strategy in Embase | 3 |
| sTable 3. Search Strategy in Cochrane | 4 |
| sTable 4.Study Quality Assessment | 5 |
| sTable 5. Results of subgroup analysis | 6 |
| sTable 6. Results of publication bias | 7-8 |
| sFigure 1. Flow diagram of study selection | 9 |

**sTable 1. Search Strategy in PubMed**

| 1. maternal |
| --- |
| 2. prepregnancy |
| 3. pregnancy |
| 4. gestational |
| 5. 1 OR 2 OR 3 OR 4 |
| 6. body mass index |
| 7. BMI |
| 8. overweight |
| 9. obesity |
| 10. anthropometry |
| 11. fatness |
| 12. body fatness |
| 13. weight gain |
| 14. adiposity |
| 15. 6 OR 7 OR 8 OR 9 OR 10 OR 11 OR 12 OR 13 OR 14 |
| 16. Asphyxia Neonatorum |
| 17. Apgar score |
| 18. birth asphyxia |
| 19. apgar score |
| 20. cord pH |
| 21. cord gases |
| 22. umbilical artery pH |
| 23. 16 OR 17 OR 18 OR 19 OR 20 OR 21 OR 22 |
| 24. 5 AND 15 AND 23 |
| 25. Limit 24 to humans |
| 26. Limit 25 to English |

**sTable 2. Search Strategy in EMBASE**

| 1. maternal.ti,ab. |
| --- |
| 2. mother?.ti,ab. |
| 3. prepregnancy.ti,ab. |
| 4. pregnancy.ti,ab. |
| 5. mother/ |
| 6. pregnancy/ |
| 7. 1 or 2 or 3 or 4 or 5 or 6 |
| 8. body mass index.ti,ab. |
| 9. BMI.ti,ab. |
| 10. overweight.ti,ab. |
| 11. obesity.ti,ab. |
| 12. anthropometry.ti,ab. |
| 13. fatness.ti,ab. |
| 14. body fatness.ti,ab. |
| 15. adiposity.ti,ab. |
| 16. body mass/ |
| 17. obesity/ |
| 18. anthropometry/ |
| 19. body fat/ |
| 20. 8 or 9 or 10 or 11 or 12 or 13 or 14 or 15 or 16 or 17 or 18 or 19 |
| 20. Asphyxia Neonatorum.ti,ab. |
| 21. Apgar score.ti,ab. |
| 22. birth asphyxia.ti,ab. |
| 23. apgar score.ti,ab. |
| 24. cord pH.ti,ab. |
| 25. cord gases.ti,ab. |
| 26. umbilical artery pH.ti,ab. |
| 27. 20 or 21 or 22 or 23 or 24 or 25 or 26 |
| 28. 7 and 20 and 27 |
| 29. Limit 24 to humans |
| 30. Limit 25 to English |

**sTable 3. Search Strategy in Cochrane**

| 1. maternal |
| --- |
| 2. prepregnancy |
| 3. pregnancy |
| 4. gestational |
| 5. 1 or 2 or 3 or 4 |
| 6. body mass index |
| 7. BMI |
| 8. overweight |
| 9. obesity |
| 10. anthropometry |
| 11. fatness |
| 12. body fatness |
| 13. weight gain |
| 14. adiposity |
| 15. 6 or 7 or 8 or 9 or 10 or 11 or 12 or 13 or 14 |
| 16. Apgar score |
| 17. birth asphyxia |
| 18. cord pH |
| 19. cord gases |
| 20. umbilical artery pH |
| 21. 16 or 17 or 18 or 19 or 20 |
| 22. 5 and 15 and 21 |

| **Table 4. The Newcastle-Ottawa Scale score of included studies** | | | | | | | | |  |
| --- | --- | --- | --- | --- | --- | --- | --- | --- | --- |
| study | Selection of exposured | Selection of non-exposed | assess of exposure | outcome | Comparability | Assess of outcome | long enough of follow up | Adequacy of follow up | |
| Nohr  2008 | 0 | 1 | 0 | 1 | 1 | 1 | 1 | 1 | |
| CHEN  2010 | 1 | 1 | 1 | 1 | 1 | 1 | 1 | 1 | |
| Ovesen  2011 | 1 | 1 | 0 | 1 | 1 | 1 | 1 | 1 | |
| Choi  2011 | 1 | 1 | 1 | 1 | 1 | 1 | 1 | 1 | |
| Marshall  2012 | 1 | 1 | 0 | 1 | 1 | 1 | 1 | 1 | |
| Raja  2012 | 1 | 1 | 1 | 1 | 1 | 1 | 1 | 1 | |
| Magann  2013 | 1 | 1 | 1 | 1 | 1 | 1 | 1 | 1 | |
| Minsart  2013 | 1 | 1 | 0 | 1 | 1 | 1 | 1 | 1 | |
| Thrift  2014 | 1 | 1 | 0 | 1 | 1 | 1 | 1 | 1 | |
| Persson  2014 | 0 | 1 | 0 | 1 | 1 | 1 | 1 | 1 | |
| Vinturache  2015 | 0 | 1 | 0 | 1 | 1 | 1 | 1 | 1 | |

| **sTable 5. results of subgroup analysis** | |  | |
| --- | --- | --- | --- |
| subgroup | Overweight (included studies) | | Obese (included studies) |
| **Gestational age** |  | |  |
| Restricted to full term infant | 1.29[1.02 1.64]; p = 0.03  ([4], [13]) | | 1.32[1.25 1.41]; p < 0.001  ([4], [13]) |
| No restriction | 1.21[1.05 1.40]; p = 0.01  ([14], [3], [15], [17], [20]) | | 1.97[1.22 3.20]; p = 0.006  ([14], [3], [15], [17], [20]) |
| **Timing of measurement of maternal BMI** |  | |  |
| Pre-pregnancy | 1.96[1.26 3.07]; p = 0.003  ([4], [3]) | | 1.97[1.22 3.20]; p = 0.006  ([4], [3]) |
| During pregnancy | 1.18[1.05 1.33]; p = 0.008  ([14], [13], [15], [17], [20]) | | 1.33[1.25 1.41]; p < 0.001  ([14], [13], [15], [17], [20]) |

| **sTable 6. Beggar's test result of publication bias** | | |  |
| --- | --- | --- | --- |
| Pooled studies | P value of Begger test | | |
|  | crude | adjust | |
| Apgar score < 7 at 1 minute |  |  | |
| underweight | 0.602 | 1.000 | |
| overweight | 0.602 | 1.000 | |
| obese | 1.000 | 1.000 | |
| Very obese | 0.602 | 1.000 | |
| Apgar score < 7 at 5 minute |  |  | |
| underweight | 0.624 | 0.806 | |
| overweight | 0.458 | 0.536 | |
| obese | 0.216 | 0.266 | |
| Very obese | 0.348 | 0.452 | |
| Apgar score < 3 at 5 minute |  |  | |
| underweight | 0.317 | 1.000 | |
| overweight | 0.317 | 1.000 | |
| obese | 0.602 | 1.000 | |
| Very obese | 0.117 | 0.296 | |
| Cord pH < 7.1 |  |  | |
| underweight | 0.317 | 1.000 | |
| overweight | 0.317 | 1.000 | |
| obese | 0.317 | 1.000 | |
| Very obese | 0.317 | 1.000 | |

**sFigure 1. Flow chart of study selection**

V

Record from literature search(N = 205)

PubMed (n= 132)

EMBASE (n= 63)

Cochrane (n=4)

additional articles (n=6)

Records for full-text screening(N= 36)

Records excluded based on title and abstract screening:

duplicates(n= 58); reviews(n= 15); other language (n= 5); intervention studies(n= 7); irrelevant(n= 86).

Records included (N= 11)

Records excluded for(N= 25):

No outcome (n= 7); Apgar score in the form of mean and SD(2); not adjusted risk (n= 13); participants overlapped(n=3)
